# Magnesium Reinforced with Inconel 718 Particles Prepared Ex Situ—Microstructure and Properties

**DOI:** 10.3390/ma13030798

**Published:** 2020-02-10

**Authors:** Zuzanka Trojanová, Zdeněk Drozd, Pavel Lukáč, Peter Minárik, Gergely Németh, Sankaranarayanan Seetharaman, Ján Džugan, Manoj Gupta

**Affiliations:** 1 Faculty of Mathematics and Physics, Charles University, Ke Karlovu 3, 121 16 Praha 2, Czech Republic; zdenek.drozd@mff.cuni.cz (Z.D.); lukac@met.mff.cuni.cz (P.L.); peter.minarik@mff.cuni.cz (P.M.); Gergely1227@gmail.com (G.N.); 2 Mechanical Engineering, National University of Singapore, Singapore 119077, Singapore; ssnseetharaman@yahoo.com (S.S.); mpegm@nus.edu.sg (M.G.); 3 ComtesFHT, Průmyslová 996, 334 41 Dobřany, Czech Republic; jdzugan@comtesfht.cz

**Keywords:** magnesium, composite, texture, mechanical properties, twinning, thermal expansion, internal friction

## Abstract

Magnesium samples reinforced with 0.7, 1.4, and 2.4 vol.% of Inconel 718 particles were prepared using a disintegrated melt deposition technique followed by hot extrusion. Mechanical properties, thermal expansion, and damping were studied with the aim of revealing the particle influence on the microstructure, texture, tensile and compressive behavior, thermal expansion coefficient, and internal friction. The flow stresses are significantly influenced by the test temperature and the vol.% of particles. A substantial asymmetry in the tensile and compressive properties was observed at lower temperatures. This asymmetry is caused by different deformation mechanisms operating in tension and compression. The fiber texture of extruded composite samples, refined grain sizes, and the increased dislocation density improved the mechanical properties. On the other hand, a decrease in the thermal expansion coefficient and internal friction was observed.

## 1. Introduction

Magnesium based microcomposites are modern advanced materials that combine a high specific modulus and strength with a significant damping capacity and dimension stability. These properties are extremely important for the car industry, where weight reduction decreases fuel consumption and CO_2_ emission. From this point of view, the design of new magnesium materials is a task of the highest priority. Magnesium micro and nanocomposites have, in the last two decades, been intensively studied. Properties of composites may be tailored for various purposes using different types of reinforcements. Mechanical properties of composites are influenced by several factors: processing technology, microstructure, substructure, the reinforcing effect of particles, and the quality of bonding between matrix and the reinforcing phase. Ceramic or metallic micro and nanoparticles introduced into Mg and Mg alloys contribute to strengthening besides of solid solution and precipitation hardening. Oxides, carbides, nitrides, or directly metallic particles have been used as the particulate reinforcements [1,2,3,4,5,6,7,8,9,10,11,12,13]. The addition of particles improved the Young modulus, hardness, mechanical properties, high temperature tensile and compressive strength, and fatigue properties. Improved corrosion, wear, and dynamic properties have also been reported. The ductility of the magnesium matrix composites was, however, adversely affected. Several review articles summarising findings on this field, together with many models describing mechanical properties of these materials, have been reported [1,2,3,4,5,6,7]. For the industrial applications of magnesium composites, physical properties such as thermal expansion, thermal conductivity, and internal friction are also important. Many composites are prepared using the powder metallurgical technologies. These materials exhibit significant plastic anisotropy following from their texture, which is a consequence of the magnesium hexagonal structure. Inconel 718 is a hardenable nickel-based superalloy designed for high temperature application. Inconel 718 also exhibits excellent strength (tensile yield stress of 1100 MPa, ultimate tensile strength of 1300 MPa) and good creep properties. This alloy has been used for jet engines and high-speed airframe parts [14]. A combination of the magnesium matrix with Inconel particles seem to be a good compromise to obtain a material with low density and improved mechanical properties. 

The main purpose of the present work consists in studying the effect of Inconel 718 particles on the mechanical thermal and anelastic properties of magnesium. A theoretical analysis of the particle influence on deformation mechanisms, strengthening, thermal expansion and internal friction is also presented. 

## 2. Materials and Methods 

Pure magnesium was reinforced with 0.7, 1.4, and 2.4 vol.% of Inconel 718 particles and was prepared in the National University of Singapore using a disintegrated melt deposition technique. Composite samples are depicted here after as Mg + 0.7 In718, Mg + 1.4 In718, and Mg+ 2.4 In718. For comparison, pure Mg samples prepared with the same technique were used. Magnesium chips were melted together with the commercially available Inconel powder (0.2–5 μm), heated up to 750 °C, and stirred for 5 min. under an argon atmosphere. The composite melt was disintegrated by a normal current-oriented argon gas to the melt stream before being deposited onto a metallic substrate (for more details see [4]). Processed ingots with a diameter of 36 mm were homogenised for 1 h at 400 °C and subsequently hot extruded to the resulting diameter of 8 mm. 

The microstructure and texture of composites were analyzed by a FEI Quanta 200 FX scanning electron microscope equipped with EDAX EBSD camera (Thermofisher Scientific, Hillsboro, OR, USA). OIM (Orientation Imaging Microscopy) software was utilized for EBSD (Electron BackScatter Diffraction) observations. The samples were first mechanically grinded and were then polished with a diamond suspension of the grade 3.1 and ¼ μm and alumina suspension of the grade 0.05 μm. Finally, the samples surface was ion-polished by Leica EM RES102 device (Leica Microsystems, Wetzlar, Germany) in order to get a high-quality surface for EBSD measurements.

The grain size was estimated using a light Olympus GX517 microscope (Olympus Corp., Tokyo, Japan) with the special Lucia (NIS-Elements 3.00) software (Laboratory Imaging, Praha, Czech Republic). Microhardness was estimated at the polished surface. For the sample preparation, the conventional mechanical polishing and etching were done using Glycol solution (1 mL HNO_3_, 24 mL water, 75 mL ethylene glycol). The microhardness of samples was measured using a Qness Q10 automatic microhardness tester (Qness GmbH, Wien, Austria) with a Vickers indenter. The indents were automatically evaluated along the line going across the sample section. The tests were performed at room temperature on the sections along extrusion direction (ED) (L orientation) and perpendicular to the extrusion direction (TD) (T orientation). The indenting load and its dwell time were 0.1 kg and 15 s, respectively. 

Tensile and compression tests were performed in an Instron 5882 universal testing machine (Instron, Norwood, MA, USA) at an initial strain rate of the order of 10^−3^ s^−1^ in the temperature interval from room temperature up to 300 °C. The samples for the tensile tests had the dog bone shape, with a gauge length of 25 mm and a diameter of 5 mm. The samples for the compression tests had a cylinder shape, with a diameter of 6 mm and 10 mm height. The load and extension/shortening were recorded with a sampling frequency of 10 Hz. The fractional change in dimension dl/l (l is the immediate length of the specimen) and the corresponding actual cross section were determined. The true stress-true plastic strain curves were calculated according the equation in [15]. We have selected these curves because they better reflect the physical base of plastic deformation. The true stresses and true strains are also used for the modelling of strain hardening processes [16]. Characteristic stresses, i.e., the tensile/compression yield stress (TYS, CYS), the stress at plastic strain ε = 0.002, the maximum stress (MTS), and compressive peak stress (CPS) were determined.

The linear thermal expansion of cylindrical samples with diameter of 6 mm and 50 mm length was measured in a protective Ar atmosphere using a modified Netzsch 410 dilatometer (Netzsch GmbH, Selb, Germany). The measurements were carried out in a wide temperature range from room temperature up to 400 °C with a heating rate of 0.9 K/min. Four thermal cycles (heating-cooling) were performed with every specimen. A permanent shortening of samples was observed after the first thermal cycle as a result of the relaxation of thermal stresses being in samples after hot extrusion. The course of following measurements was practically the same, i.e., all thermal stresses were erased in the first cycle. The physical thermal expansion coefficient (CTE), *α*, was estimated as a derivative α=(1/*L*)·(d*L*/d*T*), where *L* is the sample length. The CTE values for room temperature were estimated as an extrapolation of the CTE vs temperature curves to room temperature.

The internal friction measurements were performed at room temperature in the Resonant Frequency and Damping Analyser (RFDA, IMCE N.V., Genk, Belgium). The samples were excited to vibrations in the resonant frequency using a small striker. The damping of the sample free vibrations was registered with a microphone and was processed using special software. The resonant frequency was ~8 kHz.

## 3. Results

### 3.1. Microstructure of Samples

**Figure 1:**
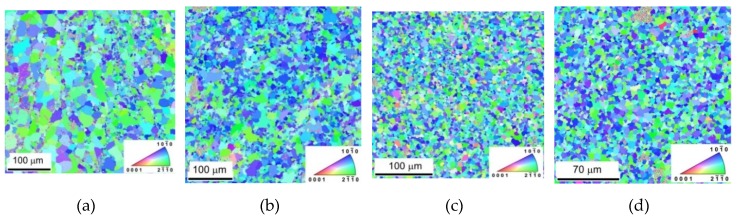
Microstructure of samples visible in sections perpendicular to the extrusion direction taken for: (**a**) pure Mg, (**b**) Mg + 0.7 In718, (**c**) Mg + 1.4 In718, and (**d**) Mg + 2.4 In718.

**Figure 2:**
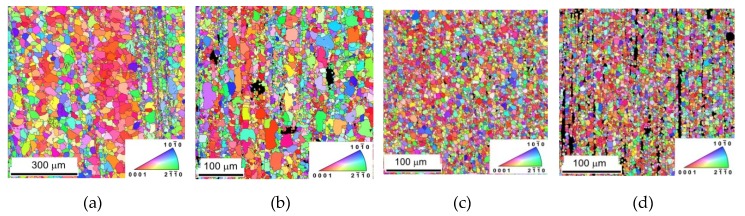
Microstructure of samples visible in sections parallel to the extrusion direction taken for: (**a**) pure Mg, (**b**) Mg + 0.7 In718, (**c**) Mg + 1.4 In718, and (**d**) Mg + 2.4 In718.

The microstructure of samples is reported in Figure 1a–d and is taken from the section perpendicular to the ED. A substantial decrease of the grain size was observed. The pictures taken from the section parallel to the extrusion direction (ED) are shown in Figure 2a–d. Small grains in rows parallel to the ED are present in Figure 2a,b. These small grains are a consequence of the partial recrystallization during the hot extrusion. The microstructure of samples with higher content of In718 particles is more uniform, as shown in Figure 2c,d. 

The dependence of grain size on the vol.% of the Inconel particles, estimated from light micrographs, is reported in Table 1. The grain size continuously decreases with increasing vol.% of particles. This decrease is marginal when the particles’ percentage increases from 1.4 to 2.4 vol.%. The grain size decreases with increasing number of nuclei which comprise the reinforcement particles. 

**Figure 3:**
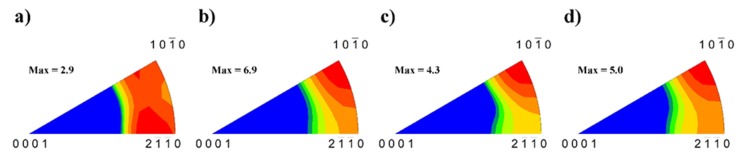
Electron back scatter (EBSD) inverse pole figures (IPF) calculated from Figure 1 for: (**a**) pure Mg, (**b**) Mg + 0.7 In718, (**c**) Mg + 1.4 In718, and (**d**) Mg + In718 (scale: blue to red).

The prevailing blue colour of grains in Figure 1a–d and the red colour in Figure 2a–d indicate existing texture of extruded samples. The EBSD pole figures calculated from Figure 1 are shown in Figure 3a–d. A typical $\left\{01\bar{1}0\right\}$ fibre texture, with the majority of grains having their basal planes parallel to the extrusion direction, was developed. It is also worth noting that, in the pure Mg sample $\left\{11\bar{2}0\right\}$, fibre is present, but Inconel addition caused significant suppression of this texture element and an increase in the strength of the $\left\{01\bar{1}0\right\}$ fibre.

The microhardness measured on the section perpendicular to the ED (L) and the section parallel to the ED (T) are reported in Table 1 together with the grain size data. Microhardness increases with the vol.% of particles. Differences between values obtained for both directions are marginal. 

### 3.2. Stres–Strain Curves

**Figure 4:**
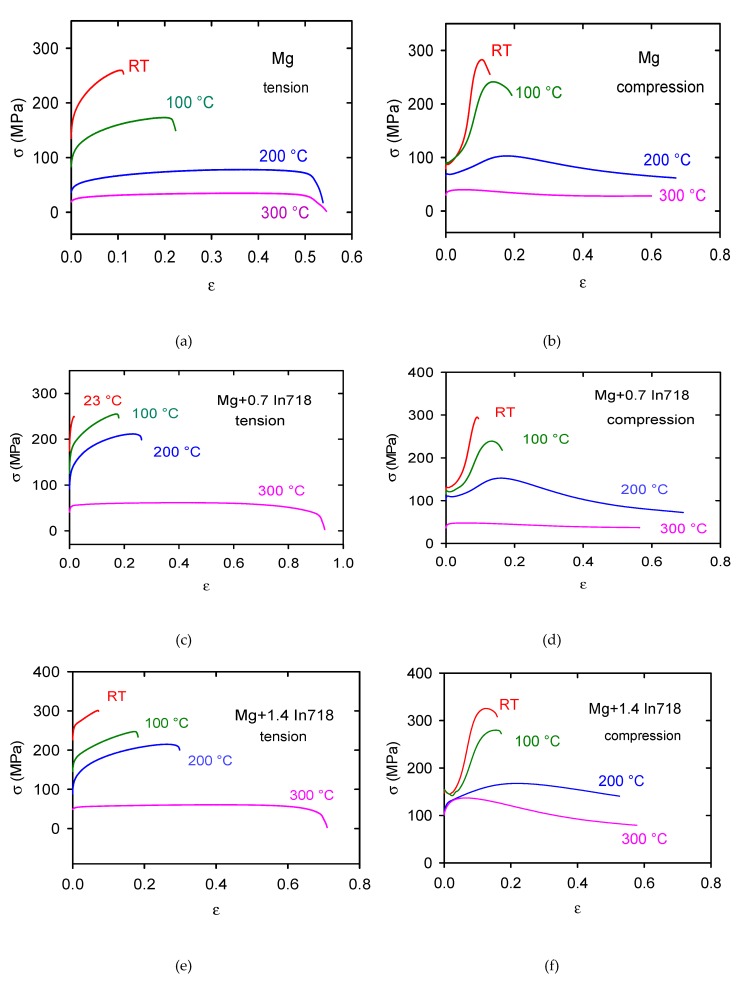

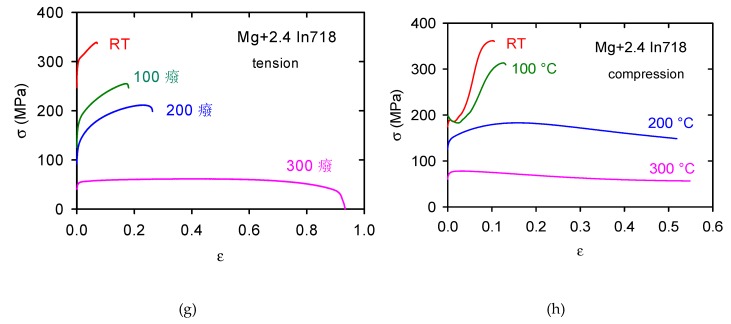
True stress–true strain curves obtained in tension and compression for (**a**,**b**) pure Mg, (**c**,**d**) Mg + 0.7 In718, (**e**,**f**) Mg + 1.4 In718, (**g**,**h**) Mg + 2.4 In718.

The true stress–true strain curves obtained in tension for pure Mg and composites with increasing volume content of In718 particles and various temperatures are shown in Figure 4a,c,e,g. All curves have a nearly parabolic character; deformation stresses decrease with increasing deformation temperatures while strains to fracture increase. 

**Figure 5:**
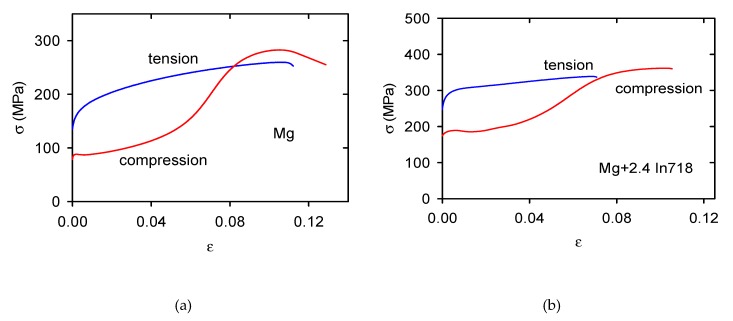
Stress–strain curves obtained in tension and compression at room temperature for (**a**) pure Mg and (**b**) Mg + 2.4 In718. The tension–compression asymmetry is obvious.

The stress–strain curves obtained in compression tests (see Figure 4b,d,f,h) exhibit a different character. For lower temperatures (RT-200 °C) the curves have the S-shape with a local stress maximum at strains 0.1–0.2. The tension–compression asymmetry of the flow curves is better to see in Figure 5a for pure Mg and Figure 5b for the Mg + 2.4 In718 sample. All curves were obtained at room temperature. 

**Figure 6:**
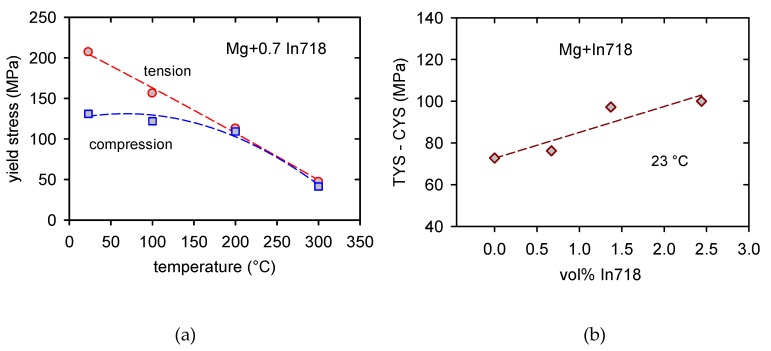
Tension–compression asymmetry: (**a**) TYS and CYS estimated for Mg + 0.7 In718 at various temperatures and (**b**) the difference between TYS and CYS estimated at room temperature depending on the vol.% of Inconel particles.

The tension–compression asymmetry decreases with increasing temperature and at about 200 °C vanishes, as is documented in Figure 6a for Mg + 0.7 In718. The tension compression asymmetry, i.e., the difference between TYS and CYS, increases with an increasing volume fraction of Inconel particles, as shown in Figure 6b. The values of TYS and CYS were estimated at room temperature. The difference between TYS and CYS is high; it increases from 73 MPa for pure Mg up to 100 MPa for Mg + 2.4 In718. 

**Figure 7:**
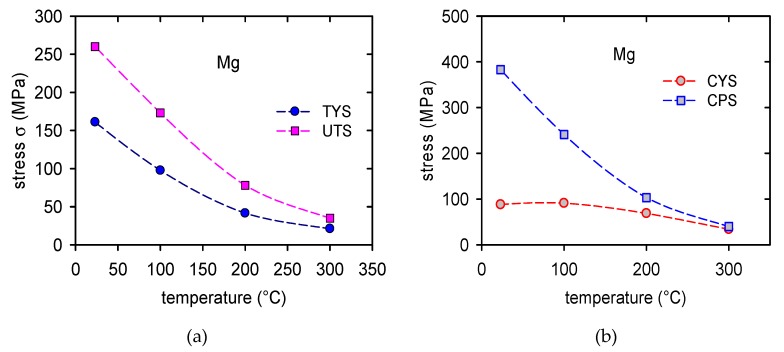
Temperature dependence of characteristic stresses obtained in (**a**) tension and in (**b**) compression for pure Mg.

**Figure 8:**
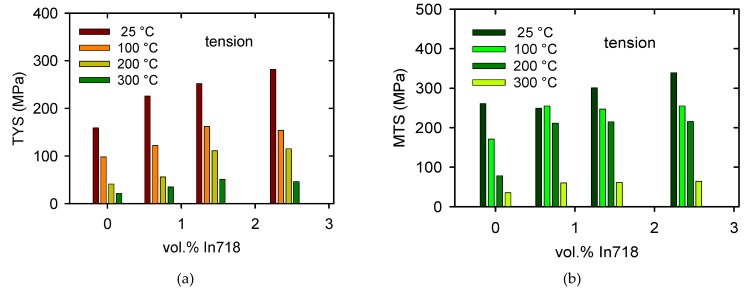
Characteristic stresses estimated for various particles’ content and temperatures in tension: (**a**) tensile yield stress (TYS) and (**b**) maximum tensile stress (MTS).

**Figure 9:**
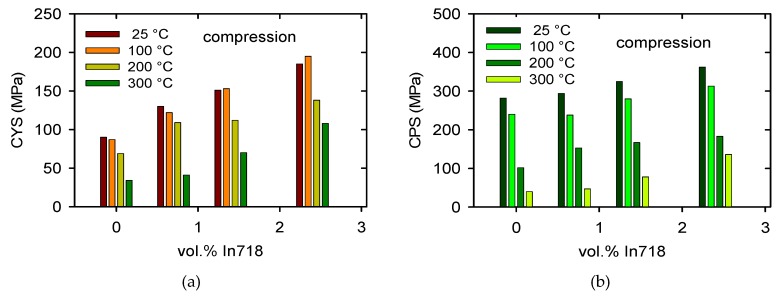
Characteristic stresses estimated for various particles content and temperatures in compression: (**a**) compression yield stress (CYS) and (**b**) compressive peak stress (CPS).

The temperature dependences of the TYS/CYS and MTS/CPS estimated for pure Mg are shown in Figure 7a,b. It is obvious that while the TYS and MTS decrease continuously with temperature, the CYS remains nearly constant at lower temperatures and then slowly decreases with temperature. The TYS and MTS estimated for all samples and temperatures are shown in Figure 8a,b, and the CYS and CPS are shown in Figure 9a,b. The TYS values estimated at room temperature increase with increasing volume fraction of In718 particles. At higher temperatures, TYS values slightly decreased for Mg + 2.4 In718. In compression tests the CYS estimated at 100 °C is slightly higher than the CYS measured at room temperature (RT) for Mg + 1.4 In718 and Mg + 2.4 In718. While in pure Mg, MTS rapidly decreases for temperatures higher than 100 °C, in samples containing In718 particles thermal stability was improved. The MTS values of composites at 200 °C were still higher than 200 MPa.

### 3.3. Physical Properties

**Figure 10:**
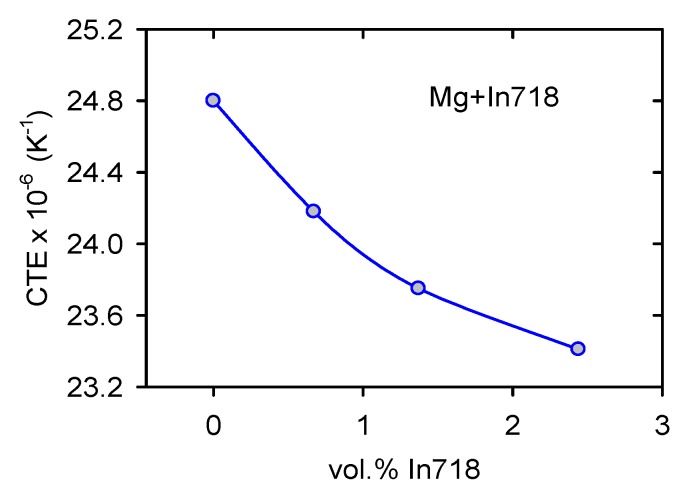
Concentration dependence of the thermal expansion coefficient (CTE).

The thermal expansion coefficient (CTE) extrapolated to room temperature is shown in Figure 10. It may be seen that CTE continuously decreases with increasing In718 content. 

**Figure 11:**
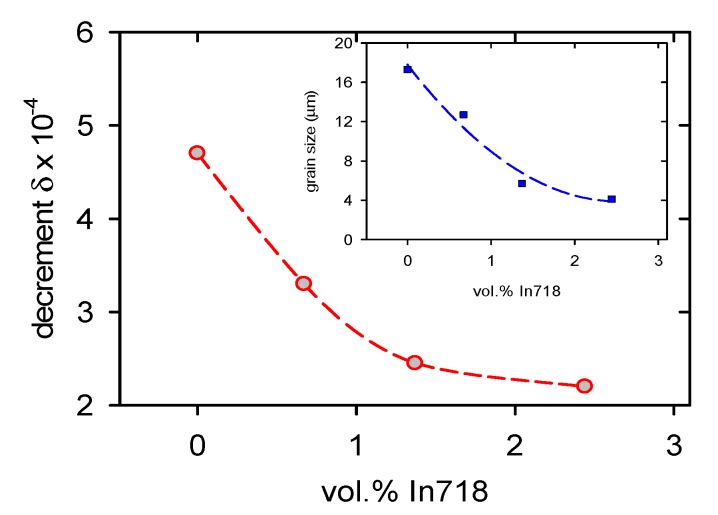
Concentration dependence of logarithmic decrement.

Similarly, the concentration dependence of the logarithmic decrement reported in Figure 11 decreases with an increasing volume fraction of the In718 particles. For comparison, the estimated decrease of the grain size is shown in the insert in Figure 11. 

## 4. Discussion

### 4.1. Mechanical Properties

**Figure 12:**
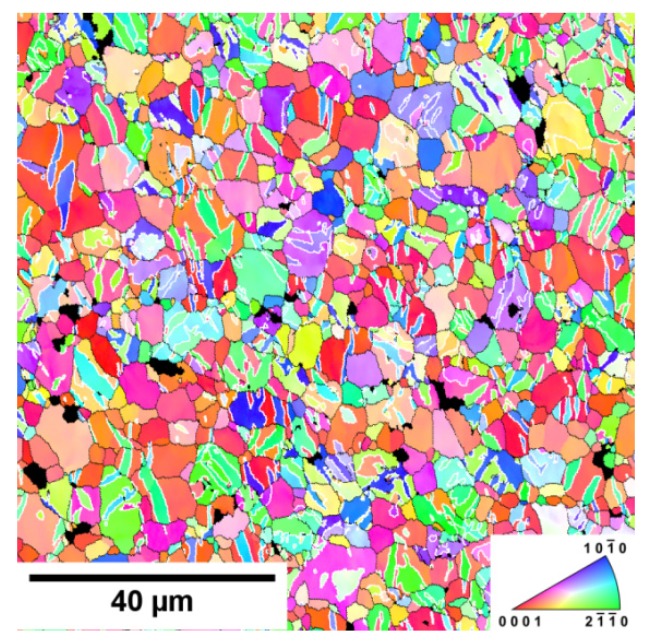
EBSD image of the Mg + 1.7 In718 sample after predeformation ε_pl_ = 0.02.

The main deformation mode in hexagonal magnesium is slip of <a> dislocations in basal (0001) planes. To satisfy the von Mises criterion for compatible deformation, which requires deformation in five independent slip systems, activity in the non-basal slip system or twinning is necessary. The EBSD pole figures shown in Figure 3 indicate that basal planes are mostly parallel to the extrusion direction (ED) meaning that the <c> axis is mostly perpendicular to the tensile/compression direction. The Schmid factor for basal planes is very low (or zero). Nevertheless, from Figure 3, it follows that basal planes are not strictly parallel to the ED. Then, we may consider that the key deformation mode at the beginning of the plastic flow during tensile deformation is the basal slip of <a> dislocations with the Burgers vector of $1/3\langle 11\bar{2}0\rangle$. These dislocations are also movable in prismatic planes. The hard orientation of prismatic planes is in the case when $\left[11\bar{2}0\right]$ direction is perpendicular to the ED [17]. The soft orientation of the prismatic slip is realized when prismatic planes are oriented ±30° to the extrusion direction. The activity of the prismatic slip $\left\{10\bar{1}0\right\}\;\langle \bar{1}2\bar{1}0\rangle$ should move the load direction always towards to the double prismatic slip (in the stereographic projection). The acoustic emission measurements show that mechanical twinning plays an important role at the tensile (and compression) deformation of textured magnesium materials. Trojanová et al. studied acoustic emission (AE) during tensile and compressive deformation of an AZ31 magnesium alloy submitted to rotary swaging [18]. They estimated massive acoustic emission at the beginning of deformation. The texture of the swaged alloy was like that observed in Mg + In718 samples. The emission signal was generated during formation of tensile twins. Such behaviour was observed by several authors and it is typical for hexagonal magnesium materials [19,20,21]. The commonly observed twinning modes are the $\left\{10\bar{1}2\right\}$ $\langle \bar{1}011\rangle$ extension twins and the $\left\{10\bar{1}1\right\}$ $\langle 10\bar{1}2\rangle$ contraction twins. The twins’ formation reorients the basal planes by 86.3°, and a compression twinning, which was observed in the later stages of deformation, by 56.2°, with respect to the parent lattice [22,23], was also observed. The Schmid factor for basal planes is, in the reoriented planes, still very low, and a slip in these planes is rather inadequate. The stress necessary for plastic deformation via prismatic slip is relatively high because of the high critical resolved shear stress (CRSS). The flow stress necessary for plastic deformation in prismatic planes is at low temperatures higher than that necessary for plastic flow in the basal system [24,25]. With increasing temperature, the critical resolved shear stress (CRSS) in the prismatic system with the <a> and <c+a> dislocations and the Burgers vector of $1/3\langle 11\bar{2}3\rangle$ rapidly decreases, and so does the difference between the TYS and MTS (see Figure 7). The true stress–true strain curves observed in compression experiments at 23 and 100 °C have an S-character with a rapid increase of strain hardening and a local maximum at strains between 0.1–0.2. Moreover, the CYS values are lower compared with the values estimated in the tensile tests. This behaviour was observed for all materials studied. The AE measurements highlighted massive twinning at the beginning of the compressive deformation in textured samples if deformed in the ED. Figure 12 shows an EBSD picture of Mg + 1.7 In718 sample predeformed in compression to 2% of plastic strain. Twin boundaries are indicated with the white colour, grain boundaries with the black one. The image was taken from the section parallel to the ED. Compared to Figure 3c, the increased representation of prismatic planes can be seen as a consequence of the basal planes’ reorientation in the twinning process.

Máthis et al. studied the twinning process in pure Mg with randomly oriented grains using in situ methods AE and neutron diffraction [26]. They developed a new method that is capable to distinguish the activity of basal and non-basal slip and twinning. They estimated that, in the stress region of 15–40 MPa in tension and 10–30 MPa in compression, the twinning deformation mode dominates. In compression, the AE activity of twinning was close to zero above 30 MPa, which simply reflects the fact that the AE is not sensitive to twin growth. This behaviour was also observed in the textured AZ31 magnesium alloy, and the twins’ growth was established by means of EBSD [18]. Hong et al., who were studying twinning in textured magnesium polycrystals deformed perpendicular to the <c> axis, estimated that various twins’ variants are activated in tension and compression [27]. In compression, only one twin variant, or the twin pair with the highest Schmid factor, is activated. Newly created twins are parallel and promote the twin growth and consequently a rapid increase of the twinned volume. At a strain of 5–6% (depending on the grain size) the whole volume is practically twinned [18]. During tension along the <c> axis, all six twin variants are possible to activate and therefore twins are formed in various directions intersecting each other. This causes a retarding of the twins’ growth and decreases the grains volume deformed by twinning. Twinning and the twins’ growth are the main deformation modes in samples deformed in compression at room temperature and 100 °C. The total deformation realised by twinning exhibits ~6%; further deformation is realized by dislocation slip. The reoriented twinned parts of grains still have unfavourable orientation for basal slip. Then, the deformation continues due to the non-basal slip of <a> and the <c+a> dislocations in the prismatic and pyramidal planes. Mechanical twinning is not a thermally-activated process, unlike the dislocation motion. This is also the reason why observed tension–compression anisotropy of the yield stress vanishes at temperatures 200–230 °C—see Figure 6a. Because of more effective thermal activation at elevated temperatures, the stress necessary for deformation continuation is comparable, or lower, for dislocation slip and twinning. The newly created twin boundaries at the very beginning of plastic deformation and thin contraction twins that only occupy small volume represent impenetrable obstacles for dislocation motion, restricting the mean free path of dislocation and thus contributing to strain hardening, which is manifested by the high values of the work hardening coefficient rate [28,29]. 

**Figure 13:**
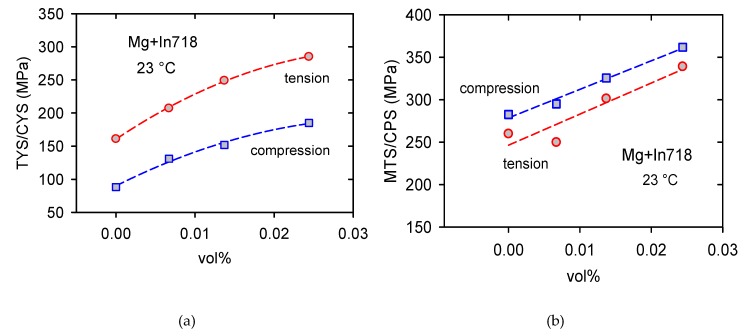
Concentration dependence of (**a**) the yield stress (TYS/CYS) and (**b**) the maximum/peak stress (MTS/CPS) estimated at room temperature.

This is also a reason explaining why the difference between CYS and CPS is, at lower temperatures, so big compared with the difference between the TYS and MTS (in the case of pure Mg ~200 MPa against ~100 MPa). This fact is well demonstrated in Figure 13.

While the yield stresses estimated in tension are higher for all concentrations than the yield stresses estimated in compression (see Figure 13a), the maximum stresses measured in compression are higher when compared with those estimated in tension (Figure 13b). This is due to a high strain hardening as a result of many twin boundaries and tiny twins, which are strong obstacles for dislocation motion. The activity of the softening processes as a cross slip and the dislocation climb is reason for the decreasing strain hardening rate observed at elevated temperatures. At the point of the maximum true stress, the work hardening rate is zero and the stable deformation process ends.

**Figure 14:**
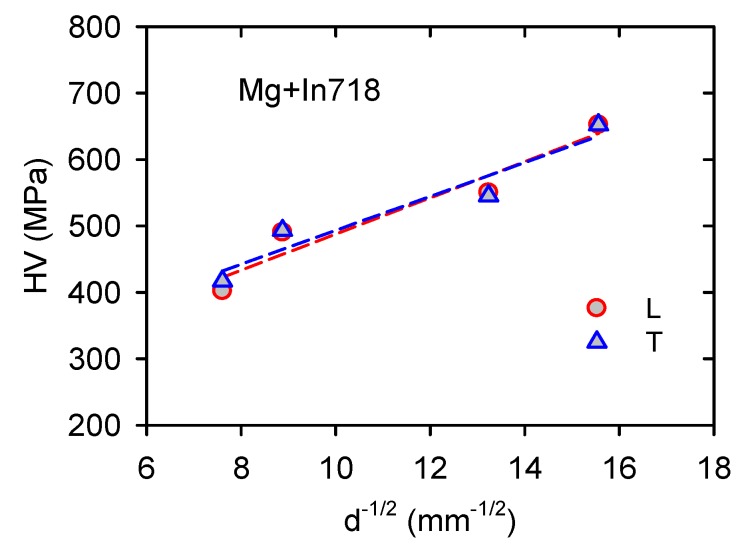
Microhardness measured in two directions plotted against *d*^−1/2^.

The grain size of samples decreases with an increasing volume fraction of Inconel particles, as is shown in Table 1. Vickers microhardness (HV) measured on the section perpendicular to the extrusion direction and on the section parallel to the extrusion direction are plotted against *d*^−1/2^ (*d* is the grain size in mm) in Figure 14. It is obvious that there is only a marginal difference between both sample sections. On the other hand, the linear course of both dependences is moderately fulfilled.

The slopes of the dependences exhibit relatively high values, i.e., *K*_H_(L) = 27.2 MPa·mm^1/2^ and *K*_H_(T) = 25.5 MPa·mm^1/2^. The small difference between HV estimated in both directions is very probably due to the fact that the microhardness measurement is, in principle, different from the plasticity experiment and therefore is not simply comparable with the tensile yield stress used, for example, in the Tabor rule [30].

### 4.2. Influence of Particles

**Figure 15:**
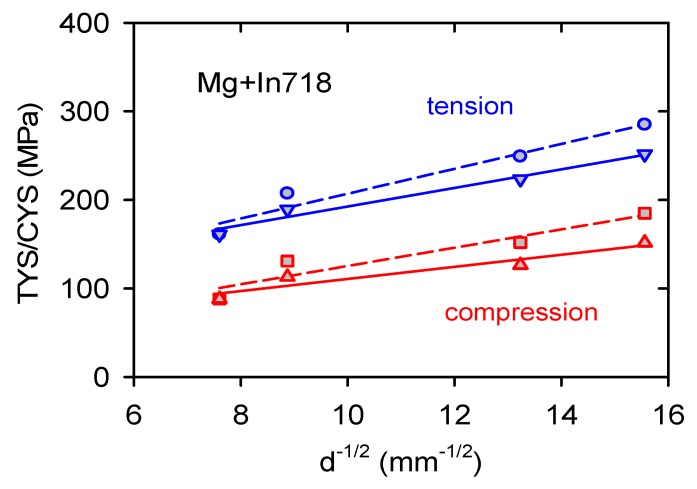
TYS and CYS plotted against *d*^−1/2^. Experimental values are linked with the dash line, and corrected values are linked with the solid line.

The experimental yield stress values obtained at room temperature were plotted against the grain size *d*^−1/2^ in Figure 15 (dash line). Two different dependences with the different slopes were found because of the tensile–compression asymmetry. The observed increase of the yield stresses with decreasing grain size represents Hall–Petch strengthening and a component reflecting the presence of Inconel particles in the magnesium matrix, Δ*σ*:

(1) $$\sigma \left(com\right)=\sigma_{0}+K_{y}d^{-1/2}+\Delta \sigma ,$$

where *σ*_0_ is a material constant denoting the stress for dislocation motion, *K_y_* is the strengthening coefficient, and *d* the average grain diameter. The strengthening contribution of particles, Δ*σ*, may incorporate several terms:

(i) Increased dislocation density due to a big difference in the CTE between the matrix and the reinforcing phase (so called thermal dislocations, *ρ_T_*);
(ii) The generation of geometrically necessary dislocations during plastic deformation, *ρ_G_*;
(iii) The load transfer of stress from the matrix to the reinforcing phase particles;
(iv) Orowan strengthening.

The generation of thermal and geometrically necessary dislocation densities, *ρ_T_* and *ρ_G_*, can be calculated according to the formulae done by Arsenault and Shi [31] and Ashby [32]:

(2) $$\rho_{T}=\frac{BVp\;\Delta \alpha \Delta T}{b\left(1-Vp\right)}\;\frac{1}{t},$$

where *B* is a geometrical constant (*B* = 12 for particles), *V_p_* is the volume fraction of particles, and Δ*α*Δ*T* is the thermal strain (Δ*α* is a difference between CTE of the matrix and particles, *b* is the Burgers vector of the newly created dislocations and the *t* minimum size of the reinforcing phase particles. Δ*T* is temperature decrease during the composite manufacture. The geometrically necessary dislocation density can be calculated according the simple relationship

(3) $$\rho_{G}=\frac{Vp8\varepsilon_{p}}{bt},$$

where *ε_p_* is plastic strain. The strengthening effect, Δ*σ_D_*, following from the increased dislocation density, may be calculated according to the known Taylor equation:

(4) $$\Delta \sigma_{D}=\alpha_{1}mGb{\left(\rho_{T}+\rho_{G}\right)}^{1/2}.$$

In Equation (4), *α*_1_ is a constant, m is the Taylor factor, and *G* is the shear modulus. The load transfer is for equiaxial particles done by the relation derived by Aikin and Christodoulou [33].

(5) $$\Delta \sigma_{LT}=0.5\;Vp\;\sigma_{y},$$

where *σ_y_* is the yield stress in the matrix. The Orowan strengthening term can be calculated as follows [34]:

(6) $$\sigma_{OR}=\frac{Gb}{\Lambda }+\frac{5}{2\pi }GVp\varepsilon_{p},$$

where *Λ* is the mean distance between particles in the slip plane. 

Because the volume fraction of particles is very low, the load transfer only plays a marginal role; the maximum value calculated for the highest concentration of 2.4 vol.% exhibits ~2 MPa. Likewise, the contribution of the Orowan term is also not weighty. Microscopic inspection revealed that reinforcing particles are situated mainly in the grain boundaries. They represent no strong obstacle for dislocation motion. From this point of view, the Orowan strengthening term is marginal. Particles in grain boundaries may prevent, at higher temperatures, the grain boundary from sliding and contributing to the higher temperature stability of the material. The main strengthening mechanism is an increase in the dislocation density. The values calculated according Equations (2) and (3) are reported in Table 2 together with the stress, Δ*σ*_D_, estimated on the base of the Equation (4). The parameters and constants used for calculations are stated in Table 3.

Subtracting the particle’s strengthening contributions, “pure” Hall-Petch term, *σ*_HP_, may be obtained. Revisited stress values corresponding to the H-P strengthening (see Table 2) were replotted against *d*^−1/2^ in Figure 14 (solid line). Experimental points were fitted by a linear dependence as: *σ*(TYS) = 87.5 + 10.5*d*^−1/2^ and *σ*(CYS) = 42.7 + 6.8*d*^−1/2^ (the slope is calculated in MPa·mm^1/2^). The grain size sensitivity of magnesium and magnesium alloys was studied in many papers depending on the grain size, texture, manufacturing method, or deformation mode [37,38,39,40,41]. Armstrong and Worthington [42] proposed that stress necessary for twinning should have similar dependence on the grain size as it was formulated for dislocation deformation.

(7) $$\sigma_{t}=\sigma_{0t}+k_{t}d^{-1/2},$$

where indices *t* is related to twinning. It is generally accepted that the grain size influences extensive twins’ formation through two aspects: (i) certain stress concentration along the grain boundary triggers the twin nucleation [22] and (ii) the grain size limits the grain growth. Owing to different deformation mechanisms in tension and compression, which are observed in textured Mg alloys, the grain size sensitivity also differs. Wang et al. [40] found for the extruded AZ31 alloy, deformed in tension parallel to the ED, *K_y_* = 9.6 MPa·mm^1/2^, which is close to the value of 10.5, that was estimated in this study. For the same AZ31 alloy extruded and deformed in compression, Chang et al. [43] found a higher value of *K_t_* = 12.3 MPa·mm^1/2^. Wang and Choo, studying the influence of texture on the Hall–Petch relationship, estimated different *K* values for various deformation mechanisms [41]. The lowest grain size sensitivity was estimated for basal slip (2.61), increased for prismatic slip (5.54), and was the highest for pyramidal slip (9.86). Extension twinning exhibits a higher value of the H-P constant *K_t_* (3.48), although the CRSS for twin nucleation is lower than the stress necessary for dislocation motion. Relatively high values of the Hall–Petch constant estimated in tension and compression indicate the importance of the non-basal slip as a deformation mechanism in textured Mg + In718 samples.

### 4.3. Thermal Expansion

Figure 11 shows a decrease in the CTE with volume fraction of In718 particles. In the literature, there are several theoretical models [44,45,46,47] expressing the concentration dependence of the reinforcing phase on the CTE. If only hydrostatic stresses exist between phases in the composite simple, Turner’s relationship for the composite CTE can be written as follows [44]:

(8) $$\alpha_{C}=\frac{\alpha_{m}V_{m}K_{m}+\alpha_{p}V_{p}K_{p}}{V_{m}K_{m}+V_{p}K_{p}},$$

where *α*, *V*, and *K* are CTE, volume fraction, and the bulk modulus, respectively, indices *C*, *m,* and *p* refer to composite, matrix, and particles, respectively. Shapery developed a new method for calculating the upper and lower bounds of the thermal expansion coefficients of isotropic and anisotropic composites with isotropic phases, and gave explicit formulas for linear coefficients of expansion, considering beside hydrostatic stresses alas well as the shear stress [46]:

(9) $$\alpha_{C}=\alpha_{m}V_{m}+\alpha_{p}V_{p}+\frac{4G_{m}}{K_{C}}\;\frac{\left(K_{C}-K_{p}\right)\;\left(\alpha_{m}-\alpha_{p}\right)\;V_{p}}{4G_{m}+3K_{p}},$$

where *G_m_* is the shear modulus of the matrix. The composite bulk modulus *K_C_* may be calculated according the Equation (10) [48].

(10) $$K_{c}=\frac{\frac{V_{p}K_{p}}{3K_{p}+4G_{m}}+\frac{V_{m}K_{m}}{3K_{m}+4G_{m}}}{\frac{V_{p}}{3K_{p}+4G_{m}}+\frac{V_{m}}{3K_{m}+4G_{m}}}.$$

**Figure 16:**
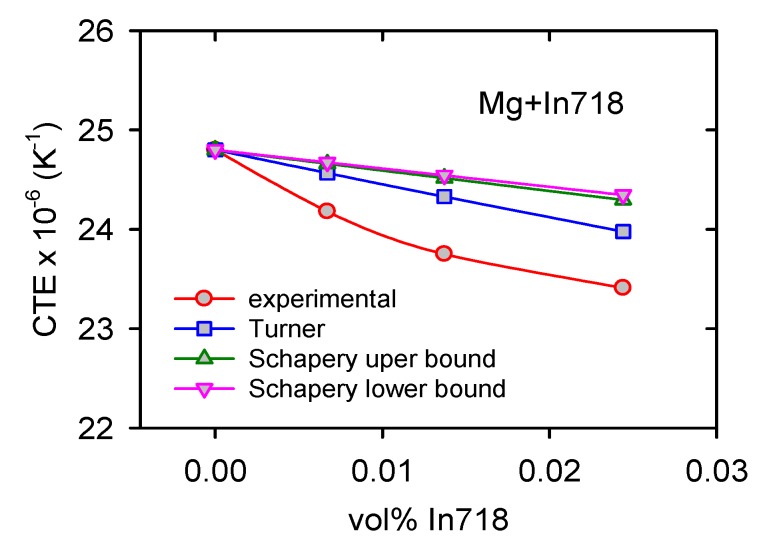
Concentration dependence of the thermal expansion coefficient.

Equation (9) expresses the situation with the upper bound. The lower bound can be calculated using the same formula when indices p and m are inverted. The composite CTEs estimated according Equations (8) and (9) are shown in Figure 16. Turner’s and Schapery’s models were used. For the calculation, the following values were used: *G_p_* = 80 GPa [47], *K_m_* = 42.5 GPa, *K_p_* = 125 GPa [49]. 

Observed decrease of the CTE is more pronounced than that predicted by the theoretical models of CTE. The decrease of experimental CTE values with the vol.% of In718 particles may be caused by two main reasons:

(i) Improved texture with increasing vol.% of In718 particles
(ii) An increase of dislocation density due to the presence of particles.

The thermal expansion of the hexagonal Mg single crystals exhibits a significant anisotropy. CTE values, estimated by the X-ray measurements of lattice constants, showed that the thermal expansion is higher in the <c> axis direction compared with that measured in the <a> direction [50].

<*a*> = 0.3208 + [7.045(T − 20) + 0.0047 × (T − 20)^2^] × 10^−6^ nm, <*c*> = 0.5276 + [11.758(T − 20) + 0.0080 × (T −20 )^2^] × 10^−6^ nm, (11)

where T is temperature in °C. Texture measurements showed that, in many grains, basal planes (containing <a> direction) are parallel to the extrusion and also the CTE measurement direction. The number of oriented grains, with the basal plane parallel to the ED, increases with the increasing vol% of particles. This improved texture decreases the thermal expansion in the ED. As it was shown experimentally in the case of Mg with 3 vol.% of BN nanoparticles, the CTE decreases after the predeformation of samples in tension [51]. An increasing dislocation density with increasing vol.% of In718 particles may cause an anomalous decrease in the CTEs. Both the reasons for the observed CTE decrease consist in the fact that the matrix microstructure is not constant, and it changes with the increasing vol.% of particles. 

### 4.4. Internal Friction

The logarithmic decrement depending on the vol.% of In718 particles is shown in Figure 11. The decrement increases with the increasing particle volume concentration. The occurrence of damping in solids may be due to a variety of reasons: the anisotropy of materials, all inhomogeneities in materials such as grain boundaries, particles of the second phases, or some internal mechanism(-s), which convert mechanical energy carried by (ultra-)sonic wave largely into heat. These internal friction mechanisms can be connected with the movement of various lattice defects [52]. Among the sources of internal friction, dislocations play an important role [53,54]. According to the Granato and Lűcke model of the dislocation internal friction, the decrement depends on the length, *ℓ*, of dislocation segments captured on weak pinning points [55,56]. Such weak pinning points may be foreign atoms or their small clusters. The periodic stress *σ* = *σ*_0_sin*ωt* (*σ*_0_ is stress amplitude and *ω* angular frequency and *t* time) is applied. For small strain amplitudes (smaller than ~10^−4^) the decrement can be expressed as:

(12) $$\delta =\frac{\Omega B\rho \omega \ell^{4}}{E_{L}b^{2}},$$

where *Ω* is an orientation factor, *B* is the coefficient of dislocation friction, *E*_L_ is tension in a dislocation line, and *b* is the Burgers vector of dislocations. The mean total density of dislocations is *ρ*. From Equation (12), it follows that the decrement is very sensitive to the dislocation substructure, which is on the other hand different in very small grains compared with the coarse-grained materials. Grain size, depending on the vol.% of particles is for the comparison introduced in the insert of Figure 11. It can be seen that the observed decrease of decrement indicates the same tendency as the decrease of the grain size. A smaller grain size limits the length of dislocation segments and the area swept in the slip plane by dislocations. The high sensitivity of this mechanism is done by the fourth power of *ℓ* in Equation (12). Collisions with phonons and electrons contribute also to the dissipation of mechanical energy. It is interesting to note that lower values of decrement, estimated in samples containing In718 particles, indicate good bonding between the magnesium matrix and Inconel 718 particles. A weak bonding between particles and the matrix should be manifested by a high internal friction because of interface sliding [57]. 

## 5. Conclusions

Magnesium reinforced by Inconel 718 particles was prepared with the disintegrated melt deposition method followed by hot extrusion. Mechanical properties, thermal expansion, and internal friction were studied. The following conclusions from this complex study can be drawn:

- Inconel 718 nanoparticles substantially refined microstructure and improve mechanical properties.
- The texture of extruded composites influenced the mechanical properties and it is also reason for the observed tension–compression asymmetry. The different deformation mechanisms operating at temperatures up to 200 °C in tension and compression were estimated.
- The main reinforcing mechanisms are Hall–Petch strengthening and an increased dislocation density.
- Texture and an increased dislocation density are very likely to be the reason for a rapid decrease in the thermal expansion coefficient with the increase in volume fraction of Inconel 718 particles.
- The observed decrease in the logarithmic decrement is caused by the grain size refinement. This also applies to the decrease in the length of the dislocation segments, which are the main source of internal friction.

## Figures and Tables

**Table 1:** Grain size and the microhardness estimated from sections perpendicular and parallel to the extrusion direction.

Sample	d (μm)	HV (L)	HV(T)
Mg	17.3 ± 8.3	40.2 ± 3.3	41.7 ± 3.1
Mg + 0.7 In718	12.7 ± 8.2	49.0 ± 3.0	49.3± 3.3
Mg + 1.4 In718	5.7 ± 2.0	55.0 ± 3.0	54.5 ± 2.4
Mg + 2.4 In718	4.1 ± 2.0	65.2 ± 4.0	65.7 ± 4.0

**Table 2:** Contribution of the increased dislocation density to the yield stress of Mg + In719 composites.

Material	*ρ_T_* (m^−2^)	*ρ_G_* (m^−2^)	Δ*σ_D_* (MPa)	*σ*_exp_ (MPa)	*σ*_HP_ (MPa)
Mg + 0.7 In718	4.6 × 10^11^	1.3 × 10^11^	17.9	131.0	113.1
Mg + 1.4 In718	9.3 × 10^11^	2.7 × 10^11^	25.4	151.8	126.4
Mg + 2.4 In718	1.6 × 10^12^	4.6 × 10^11^	33.3	185.0	151.7

**Table 3:** Constants and parameters used for calculations stress values in Table 2.

B. vector *b*	T. factor m	*α*_1_ constant	*G* modulus	CTE (Mg)	CTE (In718)	Δ*T*
3.2 × 10^−10^ m	4.6	0.35	17 Gpa	24.8	12.8	380 °C
	[35]	[36]		see Figure 10	[14]	
